# Reconstruction of the Carotenoid Biosynthetic Pathway of *Cronobacter sakazakii* BAA894 in *Escherichia coli*

**DOI:** 10.1371/journal.pone.0086739

**Published:** 2014-01-23

**Authors:** Wei Zhang, Xiaoqing Hu, Liqin Wang, Xiaoyuan Wang

**Affiliations:** 1 State Key Laboratory of Food Science and Technology, Jiangnan University, Wuxi, China; 2 Key Laboratory of Industrial Biotechnology of Ministry of Education, School of Biotechnology, Jiangnan University, Wuxi, China; 3 Synergetic Innovation Center of Food Safety and Nutrition, Jiangnan University, Wuxi, China; University of Helsinki, Finland

## Abstract

*Cronobacter sakazakii* could form yellow-pigmented colonies. However, the chemical structure and the biosynthetic pathway of the yellow pigments have not been identified. In this study, the yellow pigments of *C. sakazakii* BAA894 were purified and analyzed. The major components of the yellow pigments were confirmed as zeaxanthin-monoglycoside and zeaxanthin-diglycoside. A gene cluster containing seven genes responsible for the yellow pigmentation in *C. sakazakii* BAA894 was identified. The seven genes of *C. sakazakii* BAA894 or parts of them were reconstructed in a heterologous host *Escherichia coli* DH5α. The pigments formed in these *E. coli* strains were isolated and analyzed by thin layer chromatography, UV-visible spectroscopy, high performance liquid chromatography, and electron spray ionization-mass spectrometry. These redesigned *E. coli* strains could produce different carotenoids. *E. coli* strain expressing all the seven genes could produce zeaxanthin-monoglycoside and zeaxanthin-diglycoside; *E. coli* strains expressing parts of the seven genes could produce lycopene, β-carotene, cryptoxanthin or zeaxanthin. This study identified the gene cluster responsible for the yellow pigmentation in *C. sakazakii* BAA894.

## Introduction

Carotenoids are mainly produced by plants, and exhibit yellow, orange and red colors [1]. Because they have various biological functions, carotenoids are commercially used as food colorants, animal feed supplements and cosmetic and pharmaceutical compounds [2]. Carotenoids can also be produced by some microorganisms [3], therefore, there is an increased interest to develop microorganisms for large-scale production of carotenoids [4], [5].

Gene clusters responsible for carotenoid biosynthesis have been identified in various bacteria, and they can be divided into three types according to the gene organization [5]. The first type has the classical organization of *crtEXYIBZ*, the second type has an organization of *crtE-idi-crtXYIBZ*, and the third type has an organization of *crtE-idi-crtYIBZ*. Although different microorganisms contain the similar gene cluster, they could produce different carotenoids [5]–[8]. This might depend on their living environment, because carotenoids with different structures show different effect on membrane fluidity and thermo stability [9]. Therefore, it is interesting to investigate how bacteria synthesize different carotenoids [10].

*Cronobacter sakazakii* could form yellow-pigmented colonies, but the nature of the yellow pigments is not clear. A gene cluster responsible for the yellow pigmentation in *C. sakazakii* ES5 has been characterized [11]. This gene cluster has the organization of *crtE-idi-crtXYIBZ*. When the genes *crtE*, *crtX*, or *crtY* were inserted by transposon, *C. sakazakii* ES5 mutants became colorless [12]. However, the detailed chemical structure and biosynthesis pathway of the yellow pigments in *C. sakazakii* have not been reported.

**Figure 1:**
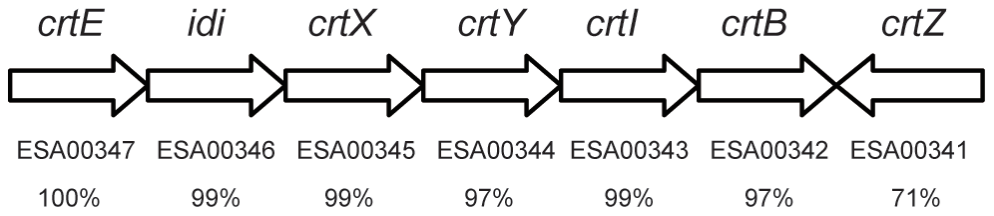
Organization of carotenoid biosynthesis genes in *C. sakazakii*. Direction of transcription is indicated by arrows. ORF numbers of the corresponding genes, and identities of the corresponding proteins in *C. sakazakii* strains ES5 and BAA894 are listed. Only the gene *idi* was annotated in the genome of *C. sakazakii* BAA894.

*C. sakazakii* BAA894 is the first genome-sequenced *C. sakazakii* strains [13]. Based on the sequence alignment, a gene cluster similar to *crtE-idi-crtXYIBZ* of *C. sakazakii* ES5 also exists in the genome of *C. sakazakii* BAA894. This gene cluster contains seven ORFs, ESA00341, ESA00342, ESA00343, ESA00344, ESA00345, ESA00346 and ESA00347, homologous to the genes *crtZ*, *crtB*, *crtI*, *crtY*, *crtX*, *idi and crtE* of *C. sakazakii* ES5, respectively (Fig. 1). In this study, the yellow pigments in *C. sakazakii* BAA894 were purified and analyzed. Some key genes in the gene cluster of *C. sakazakii* BAA894 were overexpressed in *E. coli* DH5α to explore the biosynthesis pathway of the yellow pigments. The study revealed the carotenoid biosynthesis pathway of *C. sakazakii*.

## Materials and Methods

### Bacterial strains and growth conditions

All bacterial strains used in this study are listed in Table 1. *E. coli* DH5α was used for gene expression and carotenoid production. The recombinant *E. coli* cells were cultivated for 24 hr at 37°C and 200 rpm in 50 mL Luria-Bertani (LB) medium (10 g/L trypton, 5 g/L yeast extract, 10 g/L NaCl), *C. sakazakii* BAA894was cultivated for 48 hr at 30°C and 200 rpm in 50 mL LB medium. When necessary, ampicillin was added to a final concentration of 100 mg/L.

**Table 1:** Plasmids and strains used in this study.

Plasmids and strains	Description	Resource
pWSK29	Expression vector	[27]
pWSK29-*i*	pWSK29 carrying the gene *idi*	This work
pWSK29-*Ei*	pWSK29 carrying genes *idi*, *crtE*	This work
pWSK29-*EiB*	pWSK29 carrying genes *idi*, *crtE*, *crtB*	This work
pWSK29-*EiIB*	pWSK29 carrying genes *idi*, *crtE*, *crtB*, *crtI*	This work
pWSK29-*EIB*	pWSK29 carrying genes *crtE*, *crtB*, *crtI*	This work
pWSK29-*EiYIB*	pWSK29 carrying genes *idi*, *crtE*, *crtB*, *crtI*, *crtY*	This work
pWSK29-*EiZYIB*	pWSK29 carrying genes *idi*, *crtE*, *crtB*, *crtI*, *crtY*, *crtZ*	This work
pWSK29-*EiZYIBX*	pWSK29 carrying genes *idi*, *crtE*, *crtB*, *crtI*, *crtY*, *crtZ*, *crtX*	This work
BAA894	Wild type *C. sakazakii*	ATCC
DH5α	F-, *supE*44 *Δ(lacZYA-argF) U*169 (Φ80*lacZΔM*15) *hsdR*17 *recA endA*1 *gyrA*96 *thi-*1 *relA* 1	ATCC
DH5α/pWSK29	DH5α harboring pWSK29	This work
DH5α/pWSK29-*i*	DH5α harboring pWSK29-*i*	This work
DH5α/pWSK29-*Ei*	DH5α harboring pWSK29-*Ei*	This work
DH5α/pWSK29-*EiB*	DH5α harboring pWSK29-*EiB*	This work
DH5α/pWSK29-*EiIB*	DH5α harboring pWSK29-*EiIB*	This work
DH5α/pWSK29-*EIB*	DH5α harboring pWSK29-*EIB*	This work
DH5α/pWSK29-*EiYIB*	DH5α harboring pWSK29-*EiYIB*	This work
DH5α/pWSK29-*EiZYIB*	DH5α harboring pWSK29-*EiZYIB*	This work
DH5α/pWSK29-*EiZYIBX*	DH5α harboring pWSK29-*EiZYIBX*	This work

### DNA manipulation and plasmid construction

All plasmids used in this study are listed in Table 1. All the primers used in this study are listed in Table 2. Plasmid DNA was prepared by using the EZ-10 spin column plasmid mini-preps kit from Bio Basic Inc (Markham, Canada). Restrictions enzymes and 1 kb DNA Ladder were purchased from Sangon (Shanghai, China). PCR reaction mixtures (50 µL) usually contain 5 µL 10× Ex Taq buffer, 4 µL dNTP mixture (2.5 mM each), 1 µL forward primer (20 µM), 1 µL reverse primer (20 µM), 1 µL DNA template (30 ng/µL), and 0.5 µL TaKaRa Ex Taq DNA polymerase (5 U/µL). PCR reaction was first heated to 94°C for 5 min, followed by 35 cycles of denaturation (94°C for 30 s), annealing (65°C for 30 s), and extension (72°C for 3 min). At the end, additional 10 min incubation at 72°C was used. PCR products were purified by using the TIANgel Midi purification kit from Tiangen (Beijing, China). Primers were designed according to the genomic DNA sequence of *C. sakazakii* BAA894 [13] and synthesized by Sangon.

**Table 2:** Primers used in this study.

DNA fragments amplified	Primers	Sequences	Restriction sites
*idi* (ESA00346)	*idi*-F	cgcggatccaaggagtatatacATGAAGGACAAGGAACTGAGC	*Bam*H I
	*idi*-R	agtctcgagTCATTCCTCATCCCCGACG	*Xho* I
*crtE-idi* (ESA00346-ESA00347)	*icrtE*-F	ccgagctcaggaggtatataccATGAACGCTAACGCCGTGAAAT	*Sac* I
	*icrtE*-R	gctctagaTCATTCCTCATCCCCGACGC	*Xba* I
*crtB* (ESA00342)	*crtB*-F	ggggaattcaggaggatataccATGAGTGACAAACCGCTGCT	*Eco*R I
	*crtB*-R	cggctcgagGTCTCCTTTGGTTTTCTCTACG	*Xho* I
*crtIB* (ESA00343-ESA00342)	*crtIB*-F	ggggaattcaggaggatataccATGACTAAAACTGTTGTTATCGGGTCC	*Eco*R I
	*crtIB*-R	cggctcgagCCTTTGGTTTTCTCTACGCGCC	*Xho* I
*crtE* (ESA00347)	*crtE*-F	atccgagctcAGGAGGTATATACCATGAACGCTAACGCCGTGAAATCT	*Sac* I
	*crtE*-R	accgtctagaTCAGCCAAACATAGCCAGCT	*Xba* I
*crtYIB* (ESA00344-ESA00343-ESA00342)	*crtYIB*-F	ccggaattcaaggagtatatacATGAACACGCAGTGGGATCTGATTCTCGC	*Eco*R I
	*crtYIB*-R	cggctcgagCCTTTGGTTTTCTCTACGCGCC	*Xho* I
*crtZ* (ESA00341)	*crtZ*-F	gctctagaaggaggtatataccGCCTCAGGGCGAATGGGAA	*Xba* I
	*crtZ*-R	gttgaattcAAGACCGAGAAACTGGCCC	*Eco*R I
*crtX* (ESA00345)	*crtX*-F	acgctcgagaggaggtatataccATGAGCCACTACGCCGTCAT	*Xho* I
	*crtX*-R	ccgaggtaccATCCCACTGCGTGTTCATAAG	*Kpn* I

Capital letters stand for nucleotides from chromosomal sequences. The recognition sites for restriction enzymes are underlined.

Fragments *idi*, *crtE-idi*, *crtB*, *crtIB*, *crtE*, *crtYIB*, *crtZ,* and *crtX* were individually amplified from the genomic DNA of *C. sakazakii* BAA894[13], using different primer pairs (Table 2). The forward primer usually contains a restriction site at the 5′-end and an optimized SD sequence, while the backward primer usually contains another restriction site at its 5′-end.

PCR products of *idi* and *crtE-idi* were digested with the corresponding restriction enzymes and ligated with the vector pWSK29 which was similarly digested, generating the plasmid pWSK29-*i* and pWSK29-*Ei*, respectively. PCR products of *crtB*, *crtIB* and *crtYIB* were digested with the corresponding restriction enzymes and ligated with the vector pWSK29-*Ei*, generating the plasmid pWSK29-*EiB*, pWSK29-*EiIB*, and pWSK29-*EiYIB* respectively.

PCR products of *crtE* were digested with restriction enzymes and ligated with the vector pWSK29-*EiIB* which was similarly digested, generating the plasmid pWSK29-*EIB*. PCR product of *crtZ* were digested with restriction enzymes, and ligated into the plasmid pWSK29-*EiYIB* which was similarly digested, generating the plasmid pWSK29-*EiZYIB*. PCR products *crtX* were digested with restriction enzymes, and ligated into plasmid pWSK29-*EiZYIB* which was similarly digested, generating the plasmid pWSK29-*EiZYIBX*.

These plasmids were transformed into *E. coli* DH5α, resulting *E. coli* strains DH5α/pWSK29-*i*, DH5α/pWSK29-*Ei*, DH5α/pWSK29-*EiB*, DH5α/pWSK29-*EiIB*, DH5α/pWSK29-*EIB*, DH5α/pWSK29-*EiYIB*, DH5α/pWSK29-*EiZYIB* and DH5α/pWSK29-*EiZYIBX*. Transformation of *E. coli* was performed according to the published protocol [14]. Briefly, the overnight culture of *E. coli* DH5α was inoculated into 50 mL LB media to an initial OD_600_ of 0.02, grown at 200 rpm and 37°C until OD_600_ reached 0.5. The cells were cooled on ice for 30 min, centrifuged and washed twice with ice-cold 0.1 M calcium chloride, and stored at -70°C in 1.5 mL aliquots. For transformation, aliquots of the competent cells were thawed on ice, and DNA was added. The mixture was incubated on ice for 30 min, and put in a water-bath at 43°C for 1.5 min. The mixture was then cooled on ice for 2 min, and 1 mL of LB media was added. The mixture was incubated at 37°C and rotated at 100 rpm for 1 hr, and the transformants were selected on LB agar containing ampicillin.

### Extraction of carotenoids

Bacterial cells were harvested by centrifugation at 4°C and 4000 rpm. 0.5 g of cell pellets were extracted four times with 20 mL solvent, centrifuged at 4°C and 2000 rpm to remove the cell debris. The supernatants were filtrated with 0.22 µm nylon membranes. The solvent was evaporated to get the crude carotenoids.

The extraction solvent for each carotenoid compound was determined, based on its optimum extraction efficiency after testing different solvents. Carotenoids produced by *C. sakazakii* BAA894 and *E. coli* DH5α/pWSK29-*EiZYIB* were the same, and were extracted with either cold methanol or the mixture of methanol and acetone (1∶1, v/v). Carotenoids produced by *E. coli* DH5α/pWSK29-*EiZYIB* were extracted with the cold methanol. Carotenoids produced by *E. coli* DH5α/pWSK29-EiIB were extracted with the cold petroleum ether, and carotenoids produced by *E. coli* DH5α/pWSK29-EiYIB were extracted with the mixture of hexane and petroleum ether (1∶1, v/v).

### Thin layer chromatography (TLC) analysis

The dried carotenoids was dissolved in methanol or petroleum ether, and spotted onto a silica gel 60 TLC plate. The plate was then developed in the corresponding solvents. The solvent for developing the carotenoids from *C. sakazakii* BAA894 or *E. coli* DH5α/pWSK29-*EiZYIBX* was the mixture of chloroform and methanol (65∶25, v/v); the solvent for developing the carotenoids from *E. coli* DH5α/pWSK29-*EiZYIB* was the mixture of hexane-acetone (4∶1, v/v); the solvent for developing lycopene or β-carotene was the mixture of hexane and dichloromethane (9∶1, v/v). After drying, the separation of carotenoids on the plate could be visualized.

### Purification of carotenoids

To purify the carotenoids further, preparative thin layer chromatography was employed. The colored bands on the dry TLC plates were scraped off. The silica chips were extracted with methanol for 1 hr at room temperature. The suspension was centrifuged, and the supernatant was filtrated with 0.22 µm nylon membranes and dried to get the purified carotenoids. 0.5 mL carotenoids were applied to Lichrospher C18 filler purchased from Merck. Different washing and eluting solvents were used for different carotenoids. 30% methanol was used to wash, and 75% methanol was used to elute the carotenoids produced by *C. sakazakii* BAA894 or *E. coli* DH5α/pWSK29-*EiZYIBX*; 30% methanol was used to wash, and the mixture of chloroform and methanol (1∶2, v/v) was used to elute carotenoids produced by DH5α/pWSK29-*EiZYIB*; the mixture of chloroform and methanol (1∶3, v/v) was used to wash, and the mixture of acetone and hexane (1∶3, v/v) was used to elute carotenoids produced by DH5α/pWSK29-*EiYIB* or DH5α/pWSK29-*EiIB*.

### High performance liquid chromatography (HPLC) analysis

HPLC analysis was performed using an Agilent 1100 high-pressure liquid chromatography system. The carotenoid samples were dissolved in petroleum ether, applied to a symmetry C18 column (3.9×150 mm), and eluted with a mixture of acetonitrile and dichloromethane (3∶1, v/v) at a flow rate of 1 ml/min. The carotenoids were detected at 450 nm and spectra were directly recorded online. Lycopene and β-carotene standards were purchased from Sigma (St. Louis, MO).

### Electrospray ionization mass spectrometry (ESI/MS) analysis

Dried carotenoids were dissolved in methanol and subjected to ESI/MS in the positive ion mode. The mass spectra were monitored in a range of m/z 200 to 1000 on a liquid chromatography mass spectrometry (LC/MS; Waters platform ZMD 4000) equipped with an ESI source; the parent molecular ions were further fragmented by MS/MS analysis. The evaporation temperature was 400°C, the cone voltage was 50 V, the collision voltage was 6 V and the detector voltage was 1800 V. Data acquisition and analysis were performed using MassLynx V4.1 software.

### UV-visible spectroscopy analysis

Dried carotenoids isolated from *C. sakazakii* BAA894, DH5α/pWSK29-*EiZYIB* and DH5α/pWSK29-*EiZYIBX* were dissolved in methanol, and that from DH5α/pWSK29-*EiIB* and DH5α/pWSK29-*EiYIB* were dissolved in hexane. Their absorption spectra were determined by a Shimadzu UV-visible spectroscopy.

### Structural identification of the hexose on some carotenoids

The hexoses of carotenoids were obtained by acid hydrolysis according to the published method [15] with minor modification. The carotenoids were dissolved in methanol and hydrolyzed by 2 M HCl at 80°C for 6 hr, and then the mixture was dried. The residues were dissolved in water and filtrated with a nylon membrane. The chemical structure of the hexose was determined using an ICS-5000 ion chromatography system equipped with a pulsed amperometric detector and a CarboPac PA20 column (3×150 mm, 6.5 µm).

## Results and Discussion

### Zeaxanthin-monoglycoside and zeaxanthin-diglycoside are the major components of yellow pigments produced in *C. sakazakii* BAA894

**Figure 2:**
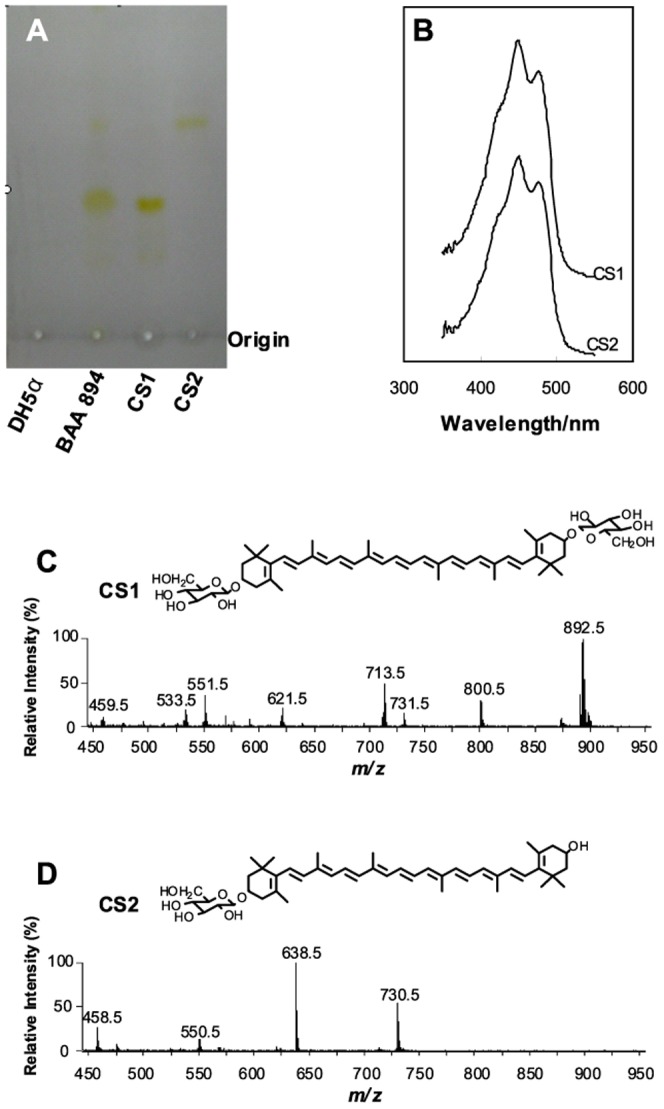
Analysis of yellow pigments produced by *C. sakazakii* BAA894. A. TLC analysis of yellow pigments CS1 and CS2 produced by *C. sakazakii* BAA894. B. UV-visible spectra of purified CS1 and CS2. C. ESI/MS analysis of the purified CS1. E. ESI/MS analysis of the purified CS2.

Yellow pigments could be formed in *C. sakazakii* BAA894 but not in *E. coli*. Two yellow substances CS1 and CS2 were extracted from *C. sakazakii* BAA894 cells (Fig. 2A). CS1 migrated slower than CS2 on TLC, suggesting that CS1 molecule is more hydrophilic than CS2. CS1 and CS2 purified by the C18 column were analyzed by UV-visible spectrometer; both showed the same absorption maxima at 450 and 478 nm (Fig. 2B), the typical peaks for carotenoids, suggesting that they belong to carotenoids [16].

Both the purified CS1 and CS2 were analyzed by ESI/MS in the positive ion mode (Figs. 2C and 2D). The major peak at m/z 892.5 in the spectrum of CS1 may be interpreted as the molecular ion [M]^+^ (Fig. 2C). The peak at m/z 800.5 could be derived from the parent ion by loss of a neutral molecule of toluene [17]. The peak at m/z 731.5 may be derived from the parent ion by loss a 161 amu moiety, suggestive of a hexose unit, and the peak at m/z 713.5 may be further derived by removal of a molecule of water. From the ion at m/z 713.5, the ion at m/z 621.5 may be derived by loss of a neutral molecule of toluene, and the ion at m/z 551.5 may be derived by loss of a glucose unit. The peak at m/z 533.5 may be derived from the peak at m/z 551.5 by removal of a molecule of water. The loss of a neutral molecule of toluene indicates the presence of extensive conjugations within the molecule CS1. The fragment pattern in the spectrum of CS1 is identical to that of zeaxanthin-diglycoside [18].

The major peak at m/z 730.5 in the spectrum of CS2 (Fig. 2D) may be interpreted as the molecular ion [M]^+^. The peak at m/z 638.5 could be derived from the parent ion by loss of a neutral molecule of toluene. The peak at m/z 550.4 may be derived from the parent ion by loss a hexane unit and a molecule of water, and the peak at m/z 458.4 may be further derived by loss of a neutral molecule of toluene. The fragment pattern in the spectrum of CS2 is identical to that of zeaxanthin-monoglycoside [18].

The results of ESI/MS analysis suggest that the yellow pigments CS1 and CS2 produced by *C. sakazakii* BAA894 are zeaxanthin-diglycoside and zeaxanthin-monoglycoside, respectively. The yield of CS1 and CS2 reached 65 µg/g and 34 µg/g of dry cell weights, respectively.

### Reconstruction of the carotenoid biosynthesis pathway of *C. sakazakii* BAA894 in *E. coli* DH5α

**Figure 3:**
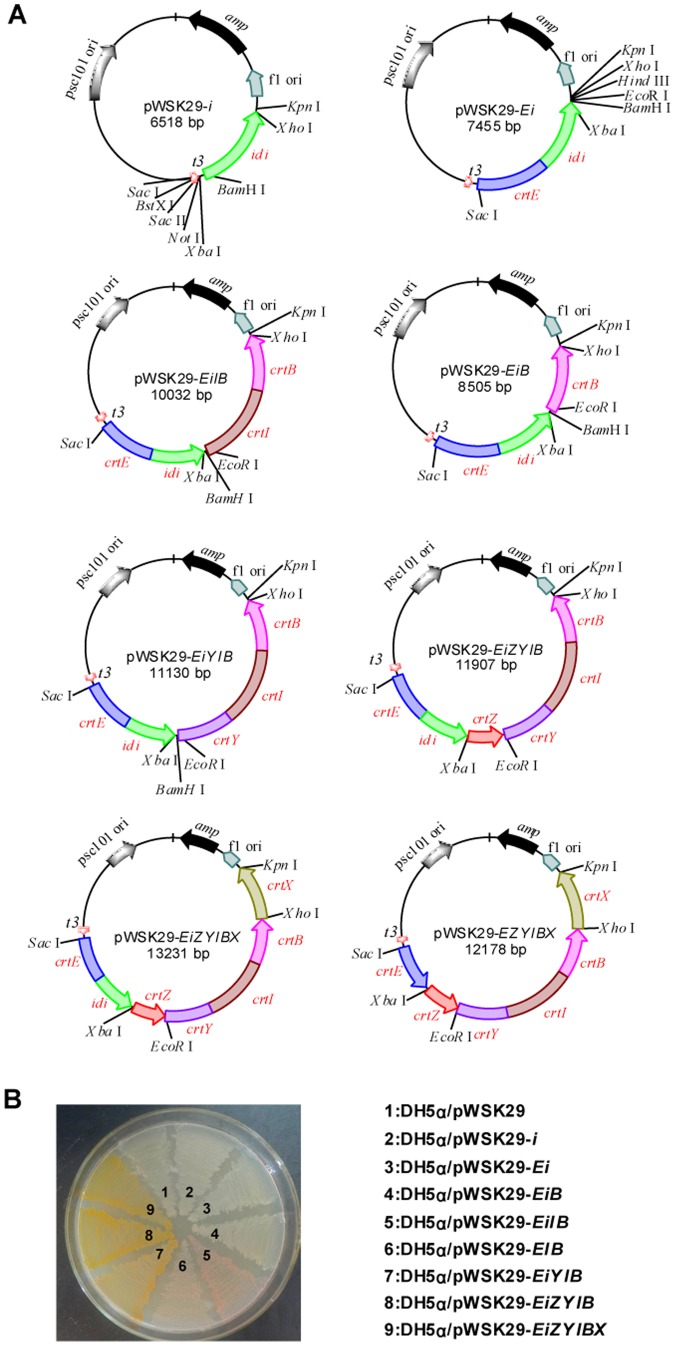
Reconstruction of the carotenoid biosynthesis pathway of *C. sakazakii* in *E. coli*. A. Seven genes *crtE*, *idi*, *crtX*, *crtY*, *crtI*, *crtB and crtZ* were amplified from the genome of *C. sakazakii* BAA894 and cloned into pWSK29 in various combinations. B. Different colors shown by cells of *E. coli* DH5α/pWSK29, DH5α/pWSK29-*i*, DH5α/pWSK29-*Ei*, DH5α/pWSK29-*EiB*, DH5α/pWSK29-*EiIB*, DH5α/pWSK29-*EIB*, DH5α/pWSK29-*EiYIB*, DH5α/pWSK29-*EiZYIB*, and DH5α/pWSK29-*EiZYIBX*.

To identify the genes responsible for the yellow pigmentation in *C. sakazakii*, A gene cluster containing seven ORFs were amplified from the genome of *C. sakazakii* BAA894 and overexpressed in *E. coli* DH5α. Based on the sequence alignment, this gene cluster of *C. sakazakii* BAA894 is similar (95% DNA homology) to the gene cluster *crtE-idi-crtXYIBZ* responsible for the yellow pigmentation in *C. sakazakii* ES5 [11]; and the seven ORFs ESA00341, ESA00342, ESA00343, ESA00344, ESA00345, ESA00346 and ESA00347 in this gene cluster are homologous to *crtZ*, *crtB*, *crtI*, *crtY*, *crtX*, *idi and crtE* in the gene cluster of *C. sakazakii* ES5, respectively. These seven ORFs or some of them were cloned into pWSK29 (Fig. 3A) and transformed in *E. coli* DH5α, forming *E. coli* strains DH5α/pWSK29, DH5α/pWSK29-*i*, DH5α/pWSK29-*Ei*, DH5α/pWSK29-*EiB*, DH5α/pWSK29-*EiIB*, DH5α/pWSK29-*EIB*, DH5α/pWSK29-*EiYIB*, DH5α/pWSK29-*EiZYIB*, and DH5α/pWSK29-*EiZYIBX*.

These *E. coli* strains grew at the similar rate, but showed different colors (Fig. 3B). *E. coli* cells expressing seven *C. sakazakii* genes *crtE*-*idi*-*crtZYIBX*, six genes *crtE*-*idi*-*crtZYIB*, or five genes *crtE*-*idi*-*crtYIB* were all yellow (Fig. 3B), suggesting these seven genes indeed related to the yellow pigmentation of *C. sakazakii* BAA894. *E. coli* cells expressing four *C. sakazakii* genes *crtE*-*idi*-*crtIB* or three genes *crtEIB* were reddish (Fig. 3B), but the former were more reddish than the latter (Fig. 3B), suggesting the existing of *idi* encoding isopentyl pyrophosphate isomerase in *C. sakazakii* could increase carotenoid titers [5]. *E. coli* cells expressing only three *C. sakazakii* genes *crtE*-*idi*-*crtB*, two genes *crtE*-*idi*, or only one gene *idi* showed no color, so did the control strain DH5α/pWSK29. These results indicate that the key genes *crtZYIX* are important for the yellow pigmentation in *C. sakazakii* BAA894. To investigate the role of the these key genes in the carotenoid biosynthesis of *C. sakazakii* BAA894, the color substances were extracted from *E. coli* DH5α/pWSK29-*EiIB*, DH5α/pWSK29-*EiYIB*, DH5α/pWSK29-*EiZYIB*, and DH5α/pWSK29-*EiZYIBX*, purified, and their chemical structures were analyzed.

### Zeaxanthin-monoglycoside and zeaxanthin-diglycoside could be synthesized in *E. coli* when seven *C. sakazakii* genes were expressed

**Figure 4:**
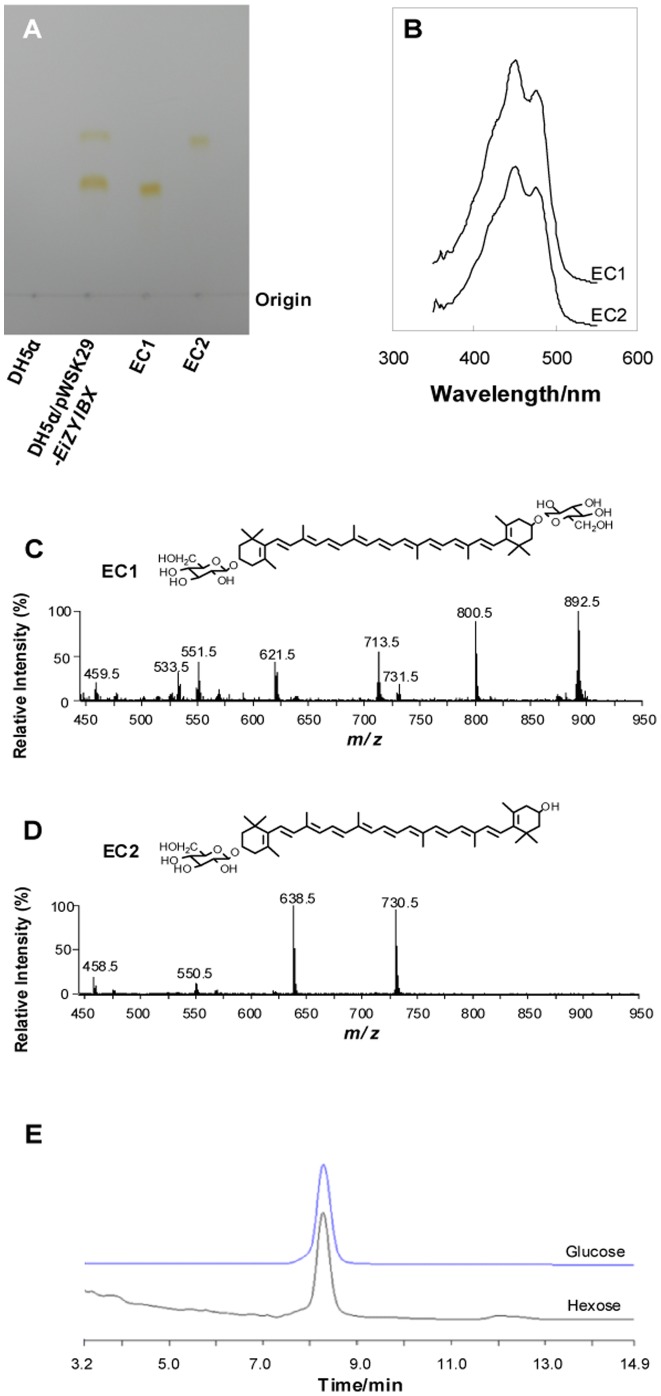
Zeaxanthin-monoglycoside and zeaxanthin-diglycoside could be synthesized in *E. coli* when seven *C. sakazakii* genes were expressed. A. TLC analysis of carotenoids EC1 and EC2 produced by DH5α/pWSK29-*EiZYIBX*. B. UV-visible spectra of purified EC1 and EC2. C. ESI/MS analysis of purified EC1. D. ESI/MS analysis of purified EC2. E. HPLC analysis of the hexose hydrolyzed from EC1.

When the gene cluster *crtE-idi-crtZYIBX* of *C. sakazakii* BAA894 were expressed in *E. coli* DH5α, two yellow substances (EC1 and EC2) were synthesized according to the analysis by TLC (Fig. 4A) and UV-visible spectrometer (Fig. 4B). Both the purified EC1 and EC2 were analyzed by ESI/MS in the positive ion mode (Fig. 4C and 4D). The pattern in the spectra of EC1 (Fig. 4C) and EC2 (Fig. 4D) are the same to that of CS1 (Fig. 2C) and CS2 (Fig. 2D), respectively. All these analysis suggest that EC1 and CS1 are the same molecule, zeaxanthin-diglycoside, and that EC2 and CS2 are the same molecule, zeaxanthin-monoglycoside. These results indicate the enzymes encoded by the gene cluster *crtE*-*idi*-*crtZYIBX* of *C. sakazakii* BAA894 function normally in *E. coli* DH5α and produce the same carotenoid molecules as in *C. sakazakii* BAA894, and suggest that the gene cluster is responsible for the production of zeaxanthin-monoglycoside and zeaxanthin-diglycoside in *C. sakazakii* BAA894. The yield of EC1 and EC2 reached 0.74 mg and 0.41 mg per gram dry cells, similar levels to the zeaxanthin-diglycoside produced by *Pantoea ananatis*. The numbers of the genes in the cluster for carotenoid biosynthesis in *C. sakazakii* BAA894 and *P. ananatis* are different; there is one more gene *idi* between *crtE* and *crtX* in the gene cluster of *C. sakazakii* BAA-894, but the amino acid sequences of the other six genes in the cluster of *C. sakazakii* BAA894 are highly homologous to those of *P. ananatis* (60.45% for CrtE, 56.38% for CrtX, 59.51% for CrtY, 81.91% for CrtI, 63.16% for CrtB, and 51.61% CrtZ). The gene *idi* of *P. ananatis* locates in other positions of the genome. The gene *idi* encodes IPP isomerase which catalyzes the crucial conversion of IPP into DMAPP, the first step controlling the overall biosynthesis of all terpenoids [19]. However, IPP and DMAPP can also be produced by isopentenyl diphosphate synthase (IDS) in MEP pathway of *C. sakazakii* BAA894 [19], [20]. The gene *idi* in *C. sakazakii* BAA894 might increase carotenoid titers, because the carotenoid yield of DH5α/pWSK29-*EiIB* (0.96 mg/g) was 60% higher than that of DH5α/pWSK29-*EIB* (0.6 mg/g).

The zeaxanthin-monoglycoside and zeaxanthin-diglycoside purified from DH5α/pWSK29-*EiZYIBX* were hydrolyzed, and the hydrolyzed hexose was analyzed by ion chromatography (Fig. 4E). The hydrolyzed hexose showed the same retention time as the standard glucose, suggesting that the hexose in both zeaxanthin-monoglycoside and zeaxanthin-diglycoside is glucose.

### Cryptoxanthin and zeaxanthin were synthesized from β-carotene

**Figure 5:**
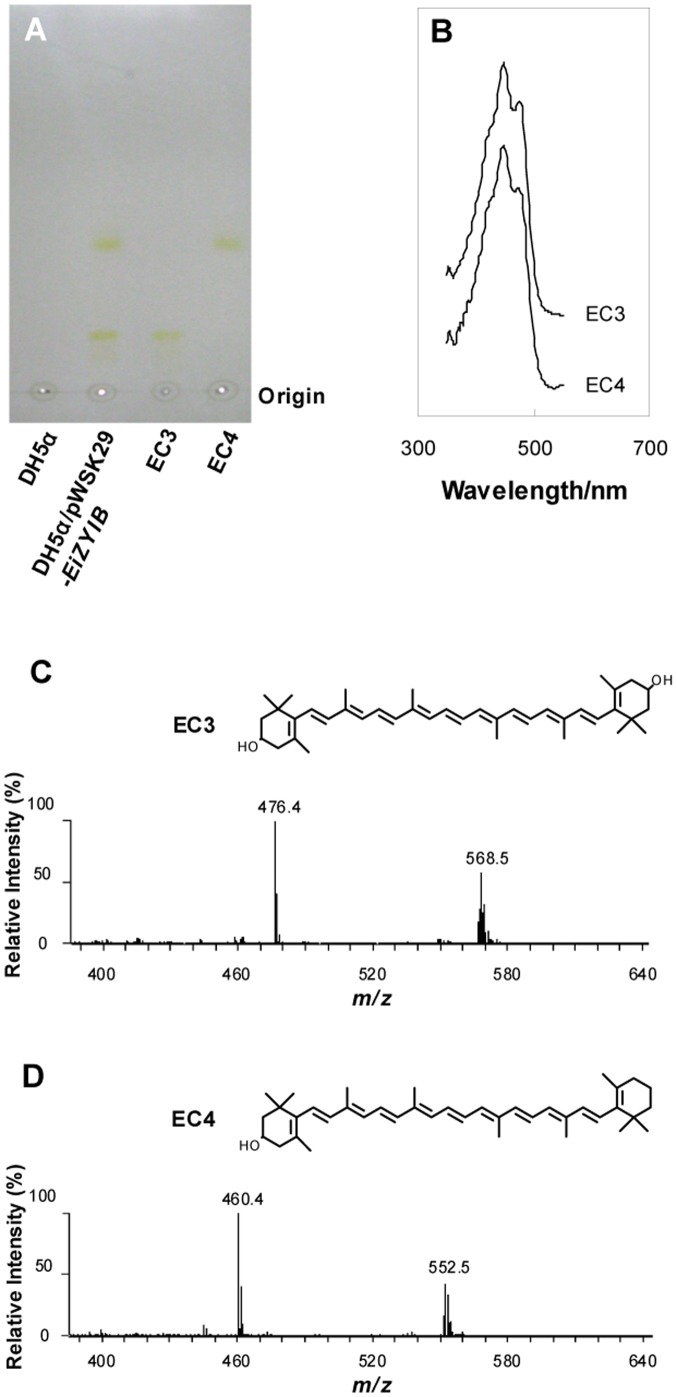
*C. sakazakii* gene *crtZ* is responsible for the biosynthesis of cryptoxanthin and zeaxanthin from β-carotene. A. TLC analysis of carotenoids EC3 and EC4 produced by DH5α/pWSK29-*EiZYIB*. B. UV-visible spectra of purified EC3 and EC4. C. ESI/MS of purified EC3. D. ESI/MS of purified EC4.

Two yellow colored substances EC3 and EC4 were extracted from *E. coli* DH5α/pWSK29-*EiZYIB*. TLC analysis showed that EC3 migrated slower than EC4 (Fig. 5A), suggesting that EC3 is more hydrophilic than EC4. Both EC3 and EC4 showed the same absorption maxima at 450 and 478 nm in the UV-visible spectrum (Fig. 5B), suggesting they have the similar chemical structures. Both the purified EC3 and EC4 were analyzed by ESI/MS in the positive ion mode (Fig. 5C and 5D). The major peak at m/z 568.4 in the spectrum of EC3 (Fig. 5C) may be interpreted as the molecular ion [M]^+^; the peak at m/z 476.4 could be derived from the parent ion by loss of a neutral molecule of toluene [17]. Similarly, the major peak at m/z 552.4 in the spectrum of EC4 (Fig. 5D) may be interpreted as the molecular ion [M]^+^, and the peak at m/z 460.4 could be derived from the parent ion by loss of a neutral molecule of toluene. Based on the molecular weight, EC3 and EC4 could be zeaxanthin and cryptoxanthin, respectively. In addition, the fragment patterns in the spectrum of EC3 and EC4 are identical to zeaxanthin and cryptoxanthin, respectively [21]. The yield of EC3 and EC4 could reach 0.57 mg/g and 0.39 mg/g of dry cell weights, respectively.

*E. coli* DH5α/pWSK29-*EiZYIB* could produce both zeaxanthin and cryptoxanthin, but DH5α/pWSK29-*EiZYIBX* could produce zeaxanthin- monoglycoside and zeaxanthin-diglycoside. This indicates that CrtX is capable of adding glycoside only to the hydroxyl of zeaxanthin, but not to the hydroxyl of cryptoxanthin.

### Lycopene or β-carotene were synthesized in *E. coli* by functional assembly of synthetic modules of *C. sakazakii* carotenoids

**Figure 6:**
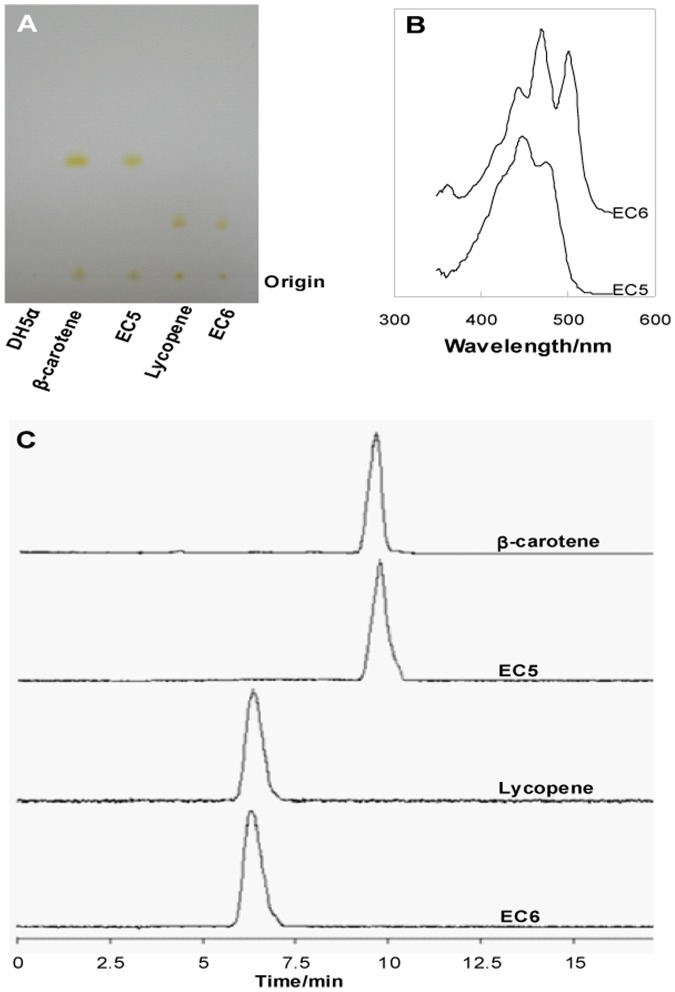
Lycopene and β-carotene could be synthesized in *E. coli* when *C. sakazakii* genes *crtE*, *idi*, *crtI* and *crtB* were expressed with or without *crtY*, respectively. A. TLC analysis of carotenoids EC1 and EC2 produced by DH5α/pWSK29-*EiIB* and DH5α/pWSK29-*EiYIB*, respectively. B. UV-visible spectra of purified EC1 and EC2. C. HPLC spectra of purified EC1 and EC2.

Only one colored substance EC5 was extracted from *E. coli* DH5α/pWSK29-*EiIB*, and another colored substance EC6 was extracted from DH5α/pWSK29-*EiYIB*. TLC analysis showed that EC6 migrated slower than EC5; EC5 has the same mobility with β-carotene, and EC6 has the same mobility with lycopene (Fig. 6A), suggesting that EC5 and EC6 could be β-carotene and lycopene, respectively. This was confirmed by the analysis of UV-visible spectroscopy and HPLC. Both EC5 and β-carotene showed the same absorption spectrum at 450 and 478 nm, while EC6 and lycopene showed the same absorption maxima at 448, 476 and 507 nm (Fig. 6B). HPLC analysis showed that EC5 and β-carotene the same retention time, so did has the EC6 and lycopene (Fig. 6C).

These results indicate that the *C. sakazakii* genes *crtE*, *idi*, *crtB*, *crtI* are responsible for the biosynthesis of lycopene; the gene *crtY* is responsible for the synthesis of β-carotene from lycopene; the gene *crtZ* is responsible for the biosynthesis of cryptoxanthin and zeaxanthin from β-carotene. The yield of EC5 and EC6 reached 0.91 mg/g and 0.83 mg/g of dry cell weights, respectively.

### The proposed carotenoid biosynthesis pathway in *C. sakazakii* BAA894

**Figure 7:**
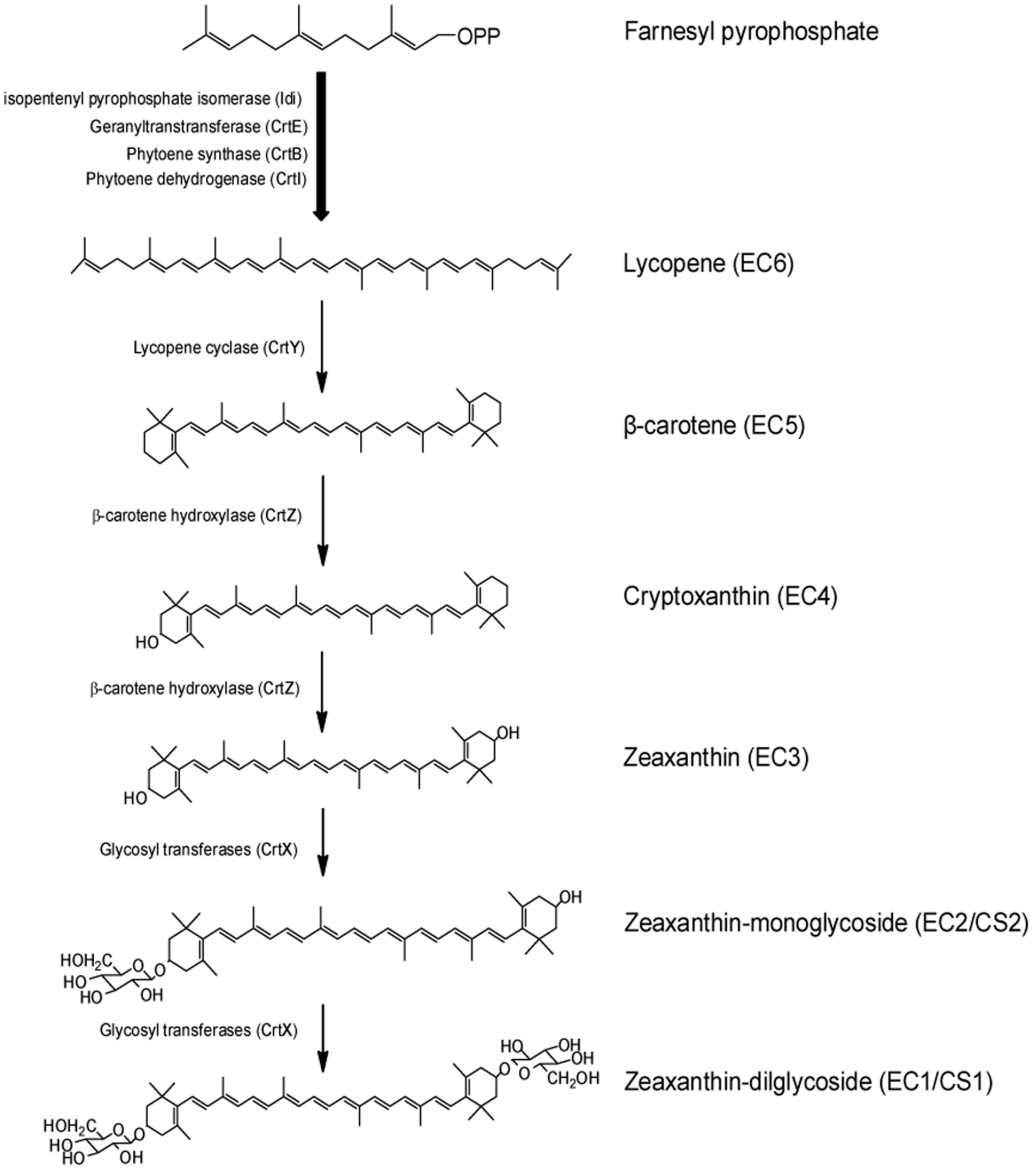
The proposed pathway of carotenoid biosynthesis in *C. sakazakii* BAA894. The names used in this article for each molecule were listed on the right.

The yellow pigments produced in *C. sakazakii* were confirmed as zeaxanthin-monoglycoside and zeaxanthin-diglycoside (Fig. 2). When the seven genes *crtE*-*idi*-*crtXYIBZ* were overexpressed in *E. coli*, the same zeaxanthin-monoglycoside and zeaxanthin-diglycoside were synthesized (Fig. 4). These results suggest that the seven-gene cluster *crtE*-*idi*-*crtXYIBZ* is responsible for the yellow pigmentation in *C. sakazakii* BAA894 (Fig. 1). Further studies were performed by expressing part of the seven gene cluster in *E. coli*, purifying the carotenoids and analyzing their chemical structures. *E. coli* expressing three genes *crtE*-*idi*-*crtB* could not produce yellow pigments (Fig. 3B). *E. coli* strains expressing four genes *crtE*-*idi*-*crtI*-*crtB*, five genes *crtE*-*idi*-*crtY*-*crtI*-*crtB*, six genes *crtE*-*idi*-*crtZYIB*, and seven genes *crtE*-*idi*-*crtZYIBX* could produce lycopene, β-carotene, zeaxanthin (or cryptoxanthin), and zeaxanthin-diglycoside (or zeaxanthin-monoglycoside), respectively (Figs. 5 and 6). Based on these results, the carotenoid biosynthesis pathway in *C. sakazakii* BAA894 was proposed (Fig. 7). The biosynthesis of the yellow pigments may start from farnesyl pyrophosphate (FPP), which is present both in *E. coli* and *C. sakazakii* [5], [11]. FPP is extended to lycopene by enzymes pyrophosphate synthase CrtE, phytoene synthase CrtB, and phytoene dehydrogenase CrtI. Lycopene is cyclized by lycopene cyclase CrtY to form β-carotene, which is changed to zeaxanthin through a hydroxylation reaction catalyzed by hydroxylases CrtZ. Glucosylase CrtX further modifies zeaxanthin to zeaxanthin-monoglycoside and zeaxanthin-diglycoside by adding one or two glucose units.

One strategy to develop the carotenoid-producing microorganisms is to reconstruct the biosynthetic pathways of carotenoids from some carotenogenic hosts in a non-carotenogenic microorganism [22]. The formation of carotenoids in *E. coli* overexpressing the genes from *C. sakazakii* indicates the flexibility of the enzymes encoded by these genes in a heterologous host. Because of the high specificity of the enzymes encoded by the genes, the end products in *E. coli* expressing the genes are usually one or two types of carotenoids. Therefore, these genes from *C. sakazakii* could be used for synthesizing carotenoids in non-carotenogenic microbes such as *E. coli* [23] and *Corynebacterium glutamicum* [24]. This would be a rational strategy for generating structurally diverse carotenoids hardly accessible in nature [25], [26].
